# Cost-effectiveness analysis of chemoradiotherapy compared to surgery in patients with stage I esophageal squamous cell carcinoma in Japan

**DOI:** 10.1007/s10147-026-03145-5

**Published:** 2026-07-23

**Authors:** Takuro Ariga, Kensuke Moriwaki, Kousuke Morimoto, Katsuyuki Sakanaka, Yoshinori Ito, Tetsuo Saito, Atsuto Katano, Satoru Matsuda, Ken Kato, Hiroya Takeuchi, Hiroshi Onishi, The Japanese Society for Radiation Oncology (JASTRO) Health Insurance Committee

**Affiliations:** 1 Department of Radiology, Nakagami Hospital, Okinawa, Japan; 2 Comprehensive Unit for Health Economic Evidence Review and Decision Support (CHEERS), Research Organization of Science and Technology, Ritsumeikan University, Kyoto, Japan; 3 Division of Health Policy and Management, Department of Biomedical Sciences, College of Life Sciences, Ritsumeikan University, Kyoto, Japan; 4 Department of Health Informatics, School of Public Health, Kyoto University Graduate School of Medicine, Kyoto, Japan; 5 Department of Radiation Oncology and Image-Applied Therapy, Kyoto University Graduate School of Medicine, Kyoto, Japan; 6 Department of Radiation Oncology, Showa University School of Medicine, Tokyo, Japan; 7 Division of Integrative Medical Oncology, Saiseikai Kumamoto Hospital, Kumamoto, Japan; 8 Department of Radiology, The University of Tokyo Hospital, Tokyo, Japan; 9 Department of Surgery, Keio University School of Medicine, Tokyo, Japan; 10 Department of Gastrointestinal Medical Oncology, National Cancer Center Hospital, Tokyo, Japan; 11 Department of Surgery, Hamamatsu University School of Medicine, Shizuoka, Japan; 12 Department of Radiology, University of Yamanashi, Yamanashi, Japan

**Keywords:** Esophageal cancer, Cost-effective analysis, QOL, ICER, Chemoradiotherapy

## Abstract

**Purpose:**

We performed a model-based cost-effectiveness analysis of the use of surgical treatment (ST) versus chemoradiotherapy (CRT) for stage I esophageal cancer in Japan.

**Materials and methods:**

A model for partitioned survival analysis was developed to estimate the long-term medical costs and quality-adjusted life years (QALYs) for patients who had received either ST or CRT. Data on survival times were taken from the JCOG0502 trial. Utility values were estimated by applying a mapping algorithm based on Japanese data from the European Organization for the Research and Treatment of Cancer Quality of Life Questionnaire. From the perspective of public healthcare, only direct medical costs were considered. A discount rate of 2% per year was applied to long-term costs and QALYs to estimate the incremental cost-effectiveness ratio (ICER) of CRT compared to ST.

**Results:**

The incremental cost of CRT versus ST was estimated to be $26,313 (USD), the incremental QALY was 0.630, and the ICER was $41,742/QALY. Sensitivity analysis showed that the parameter with a relatively large effect on the ICER was the utility weight of progressive disease with esophagus preserved patients in CRT. The variations in ICERs ranged from approximately $37,113/QALY to $47,690/QALY. With willingness-to-pay thresholds ranging from $50,000/QALY to $100,000/QALY, CRT was more cost-effective than ST, with the probability of being cost-effective estimated to range from 59.3% to 76.3%.

**Conclusions:**

In Japan, CRT for stage I esophageal cancer may be more cost-effective than ST, if the acceptable ICER is $50,000/QALY.

**Supplementary Information:**

The online version contains supplementary material available at https://doi.org/10.1007/s10147-026-03145-5.

## Introduction

According to the Japanese cancer statistics published by the National Cancer Center [1, 2], the incidence of esophageal cancer in Japan in 2019 was 20.9 cancers per 100,000 people, and the mortality was 8.9 deaths per 100,000 people. According to the data from the National Esophageal Cancer Registry of the Japanese Esophageal Cancer Association [3], the proportion of stage I (TNM 8th Edition [4]) patients is the highest incidence rate, accounting for 39.2%. The JCOG0502 study [5] by the Japan Clinical Oncology Group (JCOG) compared esophagectomy and chemoradiotherapy (CRT) for stage I esophageal squamous cell carcinoma. Results of this study showed that the overall survival (OS) rates of patients treated with CRT were non-inferior compared with surgical treatment (ST) (hazard ratio [HR] 1.052 [95% confidence interval [CI] 0.674–1.640]), however, ST obtained a longer progression-free survival (PFS) (HR: 1.478 [95% CI 1.01–2.16]). Therefore, the Japanese Guidelines for Esophageal Cancer (GL2022) [6, 7] suggest considering ST but also recommend CRT followed by appropriate monitoring and salvage therapy for patients who wish to preserve their esophagus.

In addition to OS and PFS, health-related quality of life (HRQOL) must be considered when choosing treatment modalities. There have been a few published reports on HRQOL of patients with esophageal cancer who underwent ST or CRT [8–10]. Ariga et al. [8]. showed that patients undergoing CRT had significantly better HRQOL than ST, while Chen et al. [9]. concluded that ST was better HRQOL than CRT for some of the Quality-of-Life Questionnaire (QLQ)-C30 items. Sunde et al. [10]. concluded that the advantages of ST and CRT varied depending on survey item in QLQs. Therefore, the HRQOL evaluations of patients with early esophageal cancer receiving ST vs. CRT remain uncertain.

Furthermore, evaluations from a health economics perspective have become increasingly important in recent years. Since the 1990s, health technology assessments (HTAs) and approaches to decision-making on healthcare policy have been adopted by various countries, which have used them to determine whether drugs and medical technologies can be reimbursed by public insurance and set prices [11]. In 2021, The manual for developing clinical guidelines published by The Japan Council for Quality Health Care [12] included a section on evaluating health economic. Health economic evaluations of the treatments for esophageal cancer have been performed by other countries [13–15], but there is no the cost-effectiveness of CRT compared with ST for stage I esophageal cancer based on the Japanese insurance system.

Therefore, we analyzed the cost-effectiveness and HRQOL of ST versus CRT for esophageal cancer, aiming to support treatment planning and guide healthcare policy.

## Materials and methods

### Model-based cost-effectiveness analysis

**Fig. 1:**
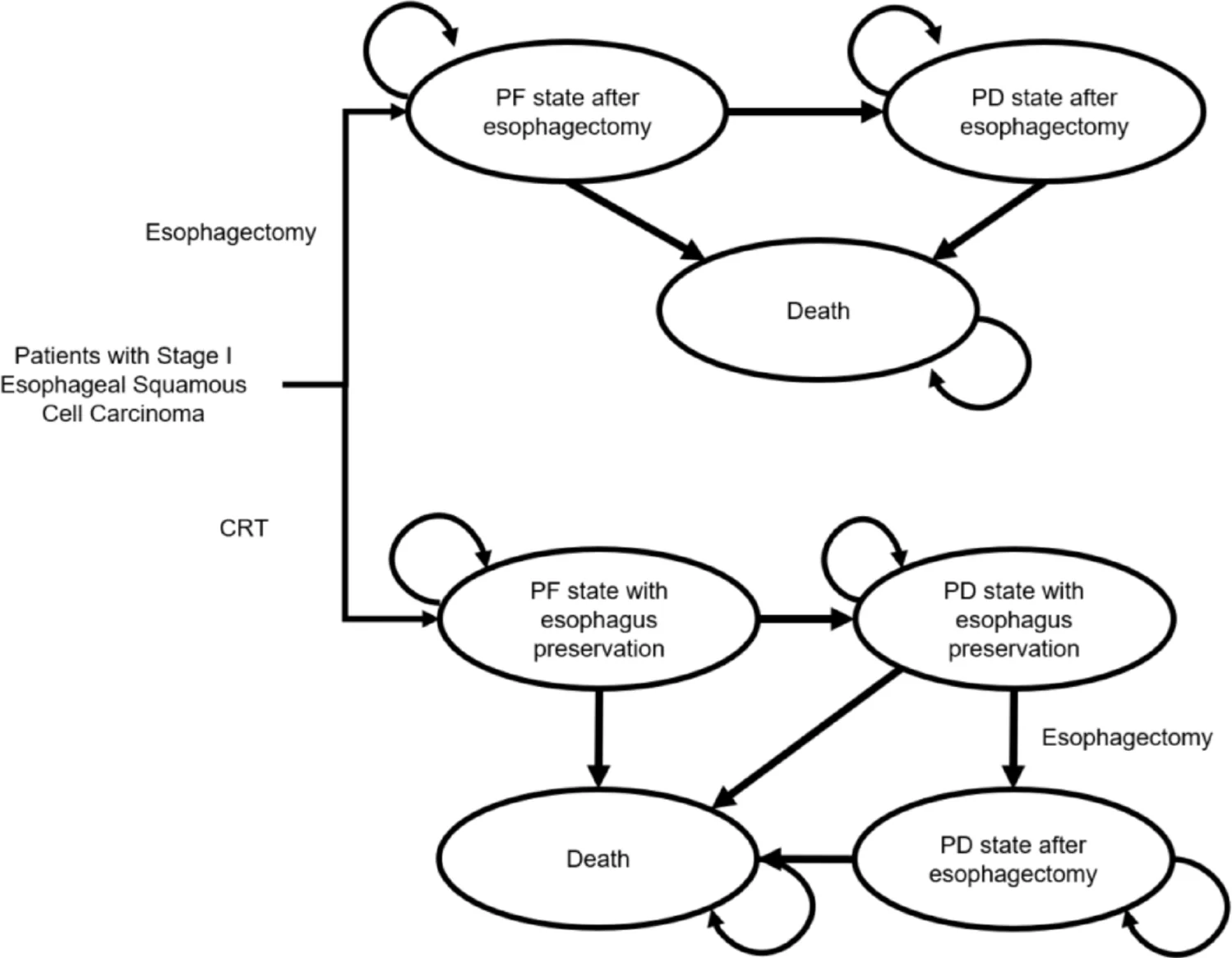
Partitioned survival analysis model. For each treatment group, each node is continuously present for an arbitrary amount of time until the status changes (curved arrows) and moves to the next node when there is a status transition (straight arrows). *Abbreviations*: CRT: chemoradiotherapy, PD: progressive disease, PFS: progression-free survival, ST: surgical therapy

A partitioned survival analysis model (PartSA) was used to assess the cost-effectiveness of CRT compared with ST. The patients who underwent ST were stratified into 3 statuses as follows: “progression-free survival (PFS) after esophagectomy”, “survival with progressive disease (PD) after esophagectomy”, and “death” (Fig. 1). Since patients who underwent CRT could survive with the esophagus preserved, the patients were stratified into 4 statuses as follows: “PFS with esophagus preserved”, “PD with esophagus preserved”, “PD after esophagectomy”, and “death” (Fig. 1). These models were used to predict long-term medical costs, duration of survival, and quality-adjusted life years (QALY) for both the ST and CRT patients.

Utility weights and cost per cycle were parameterized for each stratified patient statuses. For this analysis, parametric survival values were fitted to the Kaplan-Meier survival curves of patients in the JCOG0502 [5] that were based on esophagectomy-free survival (EFS), PFS, and OS. The parameters on the EFS, PFS, and OS Kaplan-Meyer graphs were then used to calculate changes over time in the proportions of surviving patients. The proportions and expected costs and QALYs for each stratified statuses were calculated to obtain the incremental cost-effectiveness ratio (ICER) of CRT compared to ST. ICER was expressed as “incremental cost divided by QALY”, and this represents the additional cost required for the CRT patients to gain 1 QALY greater than the ST patients.

Although there is no explicit threshold for ICER regarding cancer treatments in Japan, the Central Social Insurance Medical Council’s cost-effectiveness evaluation system operate if estimated ICERs for treating a fatal disease such as cancer range from $50,000 to $75,000/QALY (at $1 = 150 yen, 7.5 to 11.25 million yen/QALY), from $75,000 to $100,000/QALY (11.25 to 15 million yen/QALY), and greater than $100,000/QALY (15 million yen/QALY ), the adjusted portion of the official price is adjusted downward by 30%, 60% and 90% respectively. For this reason, these price adjustment thresholds were also used in this study as reference values for socially acceptable values of ICER.

The period of analysis (time horizon) was set at 40 years, and the discount rate of 2% per year was applied to the long-term costs and benefits, as based on the national cost-effectiveness evaluation guidelines [16]. The cycle length of the model was set at one month. TreeAge Pro Healthcare 2022 was used to construct and simulate the model. Ethical review was not applicable because all data used for the model and analysis were obtained from existing databases or published papers.

### Parameter settings

#### Survival curves

The PartSA model was used to extrapolate the EFS, PFS, and OS for each treatment group. Recently, Guyot et al. [17]. proposed a method to construct data on patient survival times data from images of Kaplan-Meier curves and number at-risk information by simulation. The procedure they used was as follows: data from graphs showing time vs. probability of cumulative survival times were extracted from images of Kaplan-Meier curves. Then, based on the plot data and the number at risk reported in the clinical trials, they created patient datasets.

In this study, we used the Kaplan-Meier EFS, PFS, and OS curves from the JCOG0502 [5] and fit several parametric functions to create patient datasets. We used WebPlotDigitizer (Version 4.6) to extract plot information. R (version 4.2.0) and R studio (2022.02.2 Build 485) were also used to create patient datasets. To select the optimal function, we referred to the good visual fit to the Kaplan-Meier curves of the JCOG0502 [5], and the metrics provided by the Akaike Information Criterion and Bayesian Information Criterion, which express the degree of fit. As a result, the Weibull function was judged to be the best fit, and we used this function to perform calculations in the basic analysis. We estimated outcomes by extrapolating the OS of CRT group to that of the ST group, assuming similar OS rates for both groups. When PFS and EFS intersected with OS in the long-term extrapolation, we estimated outcomes by substituting OS values for PFS and EFS in subsequent years.

#### Costs

In this model, only direct medical costs were considered from the perspective of Japanese public medical payers, and the following cost parameters were established: (i) treatment cost of ST group (/patient), (ii) medical cost for PFS after esophagectomy in the ST group (/month), (iii) medical cost for PD after esophagectomy in the ST group (/month), (iv) treatment cost of CRT group (/patient), (v) medical cost for PFS with esophagus preserved in the CRT group (/month), (vi) medical cost for PD with esophagus preserved in the CRT group (/month), (vii) medical cost for PD after esophagectomy in the CRT group (/month), and (viii) terminal care cost in both group (/patient) (Table 1).

Calculations of the above cost parameters were based on the Diagnosis Procedure Combination/Per-Diem Payment System (DPC/PDPS), a Japanese public insurance reimbursement system, and the claims database provided by the Japan Medical Data Center (JMDC) (Table 1).

**Table 1:** Parameter settings

	Estimate	SE/variability	Plausible range		Distribution	Reference
Cost parameter (USD)						
ST group						
Cost of esophagectomy including hospitalization (/patient)	20,322	± 10%	18,289	22,354	Triangle	EO
Medical cost in PFS state after esophagectomy (/month)						EO
1st year	138	± 10%	124	151	Triangle	
2nd year	123	± 10%	111	136	Triangle	
3rd year	76	± 10%	68	83	Triangle	
4th year	69	± 10%	62	76	Triangle	
5th year	62	± 10%	56	68	Triangle	
Subsequent year	55	± 10%	49	60	Triangle	
Medical cost in PD after esophagectomy (/month)	2033	30	1973	2093	Gamma	JMDC
Terminal care cost (/patient)	12,175	325	11,539	12,812	Gamma	JMDC
CRT group						
Cost of CRT including hospitalization (/patient)	7348	± 10%	6,613	8,083	Triangle	EO
Medical cost in PFS with esophagus preserved (/month)						EO
1st year	328	± 10%	295	361	Triangle	
2nd year	219	± 10%	197	241	Triangle	
3rd year	171	± 10%	154	188	Triangle	
4th year	116	± 10%	105	128	Triangle	
5th year	109	± 10%	98	120	Triangle	
Subsequent year	55	± 10%	49	60	Triangle	
Medical cost in PD with esophagus preserved (/month)	2033	30	1973	2093	Gamma	JMDC
Cost of esophagectomy including hospitalization (/patient)	20,322	± 10%	18,289	22,354	Triangle	EO
Medical cost in PD after esophagectomy (/month)	2033	30	1973	2093	Gamma	JMDC
Terminal care cost (/patient)	12,175	325	11,539	12,812	Gamma	JMDC
Utility parameter						
ST group						
PFS after esophagectomy	0.843	0.032	0.78	0.906	Beta	
PD after esophagectomy	0.768	0.032	0.706	0.83	Beta	
CRT group						
PFS with esophagus preserved	0.902	0.032	0.84	0.964	Beta	
PD with esophagus preserved	0.827	0.032	0.765	0.889	Beta	
PD after esophagectomy	0.799	0.032	0.737	0.861	Beta	
Discount rate for cost and QALY	0.02	–	0	0.04	–	
Time horizon (year)	40	–	20	40	–	

CRT: chemoradiotherapy, EO: expert opinion, JMDC: JMDC claims database, PD: progressive disease, PF: progression-free survival, QALY: quality-adjusted life years, ST: surgical treatment

The DPC/PDPS consists of two parts: the comprehensive portion, which pays a fixed fee per diem based on the diagnosis; and the piecework portion, which pays based on the treatments that were performed. The comprehensive component was defined as 33 days for the ST group and 14 days for the CRT group, in accordance with the mean duration of hospitalization as reported in the DPC/PDPS 2024.

For the ST group, a continuous hospitalization period was implemented. The surgical fee model was based on an open esophagectomy with three-field lymph node dissection, gastric tube reconstruction, and anesthesia fees. For the CRT group, the radiation fee model was based on the four-field irradiation technique, and 2 cycle chemotherapy comprised seven consecutive days of hospitalization. Outpatient irradiation fees were incorporated into the piecework portion during outpatient visits.

The JMDC claims database is Japan’s largest privately available epidemiological claims database. It accumulates subscriber registers, receipts (inpatient, outpatient, and prescription), and data from health checkups provided by insurers. The database assigns a unique ID to each subscriber, which allows the tracing of transfers and visits to multiple facilities. However, elderly patients older than 75 years are not included in the JMDC database because of variations in the different insurance medical fee systems in Japan.

In this study, during the period from January 2005 to March 2021, we selected for analysis case histories with the standard diagnostic categories that related to esophageal cancer (*N* = 62,247). The extracted data was processed into a panel format of data over time to simplify the tabulation of changes in medical costs over time.

The initial treatment costs in cost parameters (i) and iv) were based on the DPC/PDPS 2024. For the follow-up examinations in cost parameters (ii) and v), we built models based on the post-treatment survey date by Nakanoko et al. [18] and calculated based on the DPC/PDPS 2024.

The pre-death medical expenditures of patients with esophageal cancer showed that the consumption of medical resources increased two months before death, peaked in the month before death, and decreased in the month after death. We defined the total medical cost in the three months before death as cost parameter viii).

The JMDC claims database does not explicit the recurrence of cancer. We performed an exploratory simulation of the PartSA model in the base case setting and found that the duration of PD in CRT group was about 23 ~ 24 months. Therefore, we assumed that the two years before the terminal stage (4 ~ 28 months before death) was the period with PD and estimated the cost parameters iii), vi), and vii). In this analysis, the ST and CRT groups were assumed to have the same medical costs for PD and end-of-life care.

#### Utility weights

The utility weights for each status were estimated based on published data by Ariga et al. [8]. The mapping algorithm used from EORTC-QLQ30 to EQ-5D-5 L that was developed by Hagiwara et al. [19] was used to transform from EORTC-QLQ30 data to utility weights.

#### Sensitivity analysis

In this study, a one-way sensitivity analysis was conducted for the parameter settings in the model, and the results of this sensitivity analysis are summarized in a tornado diagram.

To estimate the distribution of probabilities for ICERs and to perform a probabilistic assessment of cost-effectiveness, we performed a probabilistic sensitivity analysis that was based on 1000 s-order Monte Carlo simulations. We set up probability distributions limited to those parameters of the model for which information on probability distributions as 95% CI. We repeated the calculations of expected cost, expected QALY, and ICER 1000 times by sampling the input values for each parameter from the established probability distribution. Based on results of second-order Monte-Carlo simulations, we constructed cost-effectiveness acceptability curves. We estimated the proportion of iterations in which CRT would be preferred in terms of cost-effectiveness, assuming willingness-to-pay thresholds of $50,000, $75,000, and $100,000/QALY.

As mentioned earlier, we extrapolated the OS for CRT OS to the ST group to analyze the base case. To account for the impact of OS differences, we analyzed the ICER when the OS for ST group was adjusted by plus-minus10%, with the OS for CRT group set as HR = 1.

Additionally, three scenario analyses were performed. In JCOG0502 [5], the mean age in the ST group was 3 years younger, whereas Ariga et al. [8] reported no significant difference in mean age. Therefore, an additional analysis was conducted to evaluate the ICER under the age difference observed in JCOG0502 [5]. In this scenario analysis, age- and sex-specific utility values for the Japanese general population [20] were multiplied by the utility value for each health state to assess how differences in background characteristics between the two groups affected QALYs and the ICER (scenario analysis 1). Additional scenario analyses were also conducted for follow-up costs. In the present study, a declining cost model was constructed based on the questionnaire reported by Nakanoko et al. [18]. However, compared with the follow-up protocol in JCOG0502 [5], the visit frequency in our model decreased after the third year. Therefore, an additional ICER analysis was performed using a cost assumption in which the second-year follow-up cost was extended through the fifth year to be comparable to the JCOG0502 [5] follow-up protocol (scenario analysis 2). Finally, an additional analysis was conducted to evaluate the impact of incorporating immune-checkpoint inhibitors (ICI) at recurrence. Although ICI has been reimbursed in Japan since 2018, accurately estimating ICI-related costs from the JMDC dataset used in this study remains difficult. Therefore, based on CheckMate 648 [21], we developed a cost model for a 60-kg patient with recurrent disease receiving ipilimumab and nivolumab and assessing the impact of ICI (scenario analysis 3). The parameters for the scenario analysis are shown in Supplementary Table 1.

## Results

### Base case analysis

**Fig. 2:**
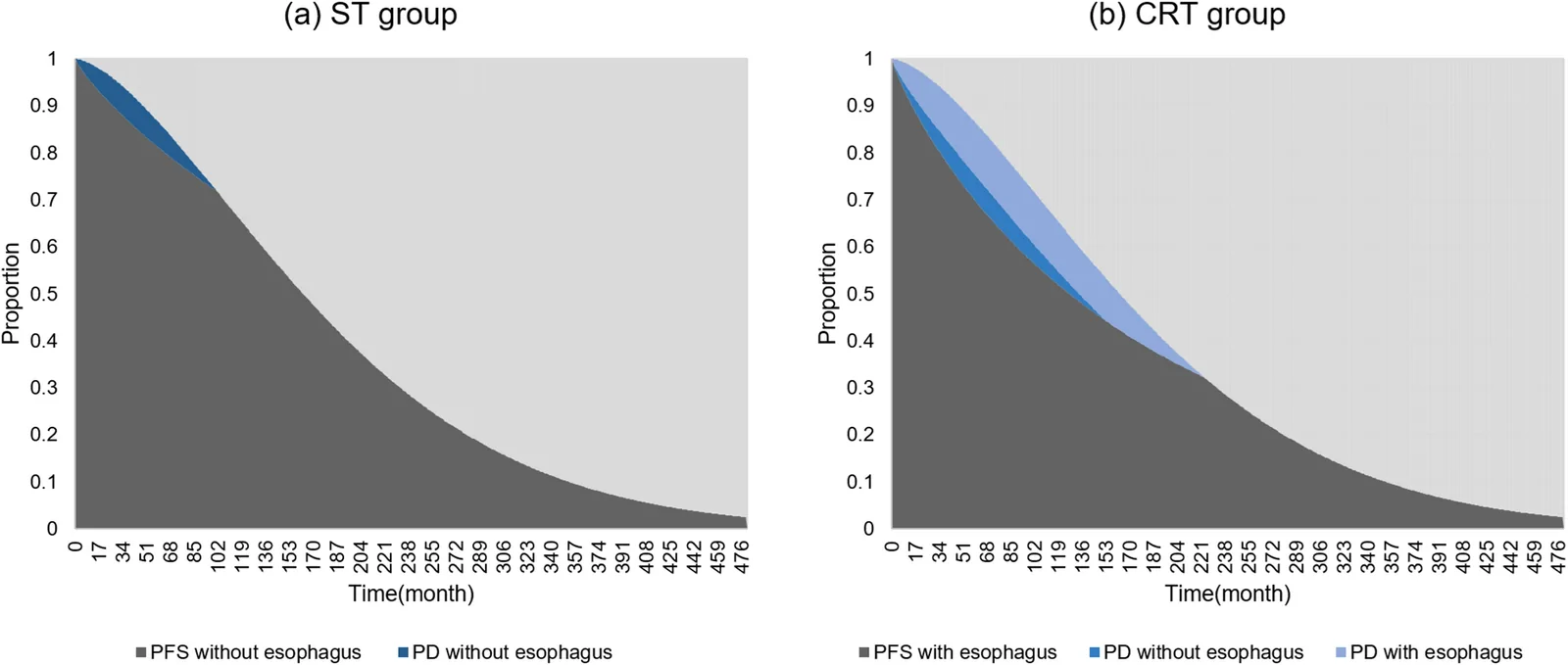
Fitting a parametric function to the Kaplan-Meier curve. The percentage of surviving patients is shown on the vertical axis of each figure. Figure 2a shows the ST group curves. The area under the curve in blue represents the mean survival time in the patients with PD, and the area under the curve in grey represents the mean time of progression-free survival. Figure 2b shows the CRT group curves. The area under the curve in light blue represents the mean survival time of patients with PD without an esophagus, the area under the curve in blue represents the mean survival time of patients with PD with an esophagus, and the area under the curve in grey represents the mean PF survival time. *Abbreviations*: CRT: chemoradiotherapy, PD: progressive disease, PFS: progression-free survival, ST: surgical therapy

The Utility weight for each status was shown in Table 1, and the results of cohort simulation on trends in health status and long-term medical costs for each group are summarized in Fig. 2; Table 2. The incremental QALY for the CRT group relative to the ST group was estimated to be 0.630. Although the initial treatment cost was lower in the CRT group, showed that the incremental total medical cost for CRT was $26,313 higher than for ST, with the ICER estimated to be $41,742/QALY.

**Table 2:** Base case results

	ST	CRT	Incremental (vs. ST)
Life years	15.323	15.323	0.000
Duration of PFS after esophagectomy (year)	14.969	0.000	− 14.969
Duration of PFS with esophagus preserved (years)	0.000	13.392	13.392
Duration of PD after esophagectomy (years)	0.354	0.444	0.091
Duration of PD with esophagus preserved (years)	0.000	1.487	1.487
QALY	10.573	11.203	0.630
Total cost (USD)	48,174	74,486	26,313
Initial treatment cost	20,163	7262	− 12,901
Medical cost during PFS after esophagectomy	10,988	0	− 10,988
Medical cost during PFS with esophagus preserved	0	16,538	16,538
Medical cost during PD after esophagectomy	8010	9669	1659
Cost of esophagectomy in CRT group	0	1066	1066
Medical cost during PD with esophagus preserved	0	30,939	30,939
ICER (USD/QALY gained)			41,742

CRT: chemoradiotherapy, ICER: incremental cost-effectiveness ratio, PD: progressive disease, PFS: progression-free survival, QALY: quality-adjusted life years, ST: surgical treatment

### Sensitivity analysis

**Fig. 3:**
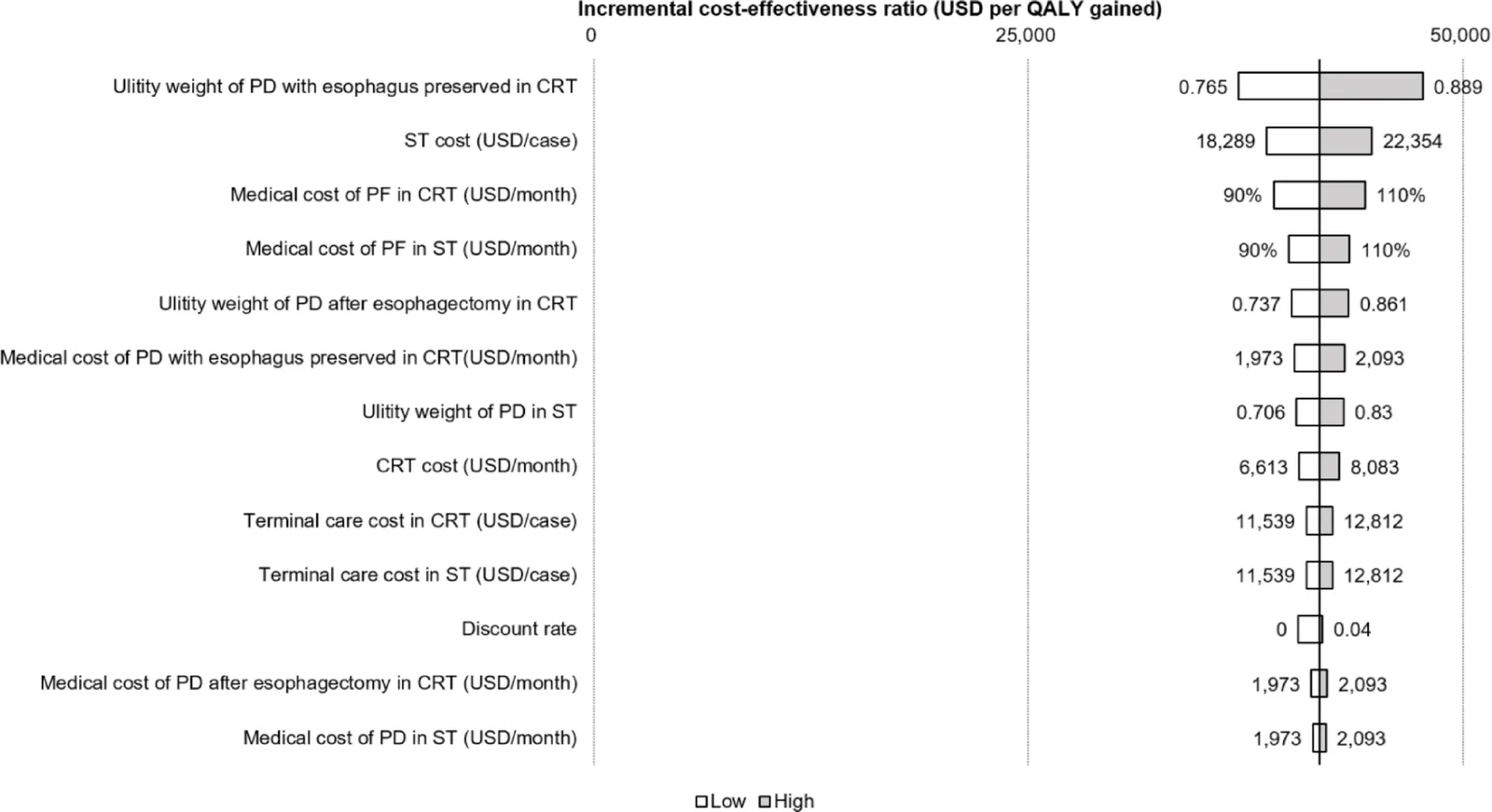
Results of the tornado diagram of the deterministic sensitivity analysis. The maximum and minimum values are appended to the bar chart for each parameter. *Abbreviations*: CRT: chemoradiotherapy, PD: progressive disease, PFS: progression-free survival, ST: surgical therapy, USD: US Dollar, QALY: quality-adjusted life years

The results of the deterministic sensitivity analysis are summarized in Fig. 3. The parameter that had a relatively large impact on the ICER in CRT was the utility weight of PD with esophagus preserved, and the variation to these parameters was in the range of $37,113/QALY to $47,690/QALY. The results suggested that ST might dominate CRT, depending on the established utility weights in PFS for the CRT and ST groups, as shown in Table 3.

**Table 3:** Sensitivity analysis of utility weights

	Treatment	Cost (USD)	Incremental cost (USD)	QALY	Incremental QALY	ICER (USD/QALY)
*Utility weight for PFS with esophaguspreserved in CRT group*						
0.840	ST	48,174		10.573		
	CRT	74,486	26,313	10.527	− 0.046	Dominated
0.871	ST	48,174		10.573		
	CRT	74,486	26,313	10.865	0.292	90,028
0.902	ST	48,174		10.573		
	CRT	74,486	26,313	11.203	0.630	41,742
0.933	ST	48,174		10.573		
	CRT	74,486	26,313	11.541	0.968	27,169
0.964	ST	48,174		10.573		
	CRT	74,486	26,313	11.879	1.307	20,139
*Utility weight for PFS in ST group*						
0.780	ST	48,174		9.801		
	CRT	74,486	26,313	11.203	1.402	18,773
0.812	ST	48,174		10.187		
	CRT	74,486	26,313	11.203	1.016	25,898
0.843	ST	48,174		10.573		
	CRT	74,486	26,313	11.203	0.630	41,742
0.875	ST	48,174		10.958		
	CRT	74,486	26,313	11.203	0.245	107,516
0.906	ST	48,174		11.344		
	CRT	74,486	26,313	11.203	− 0.141	Dominated

CRT: chemoradiotherapy, PFS: progression-free survival, ICER: incremental cost-effectiveness ratio, QALY: quality-adjusted life years, ST: surgical treatment

**Fig. 4:**
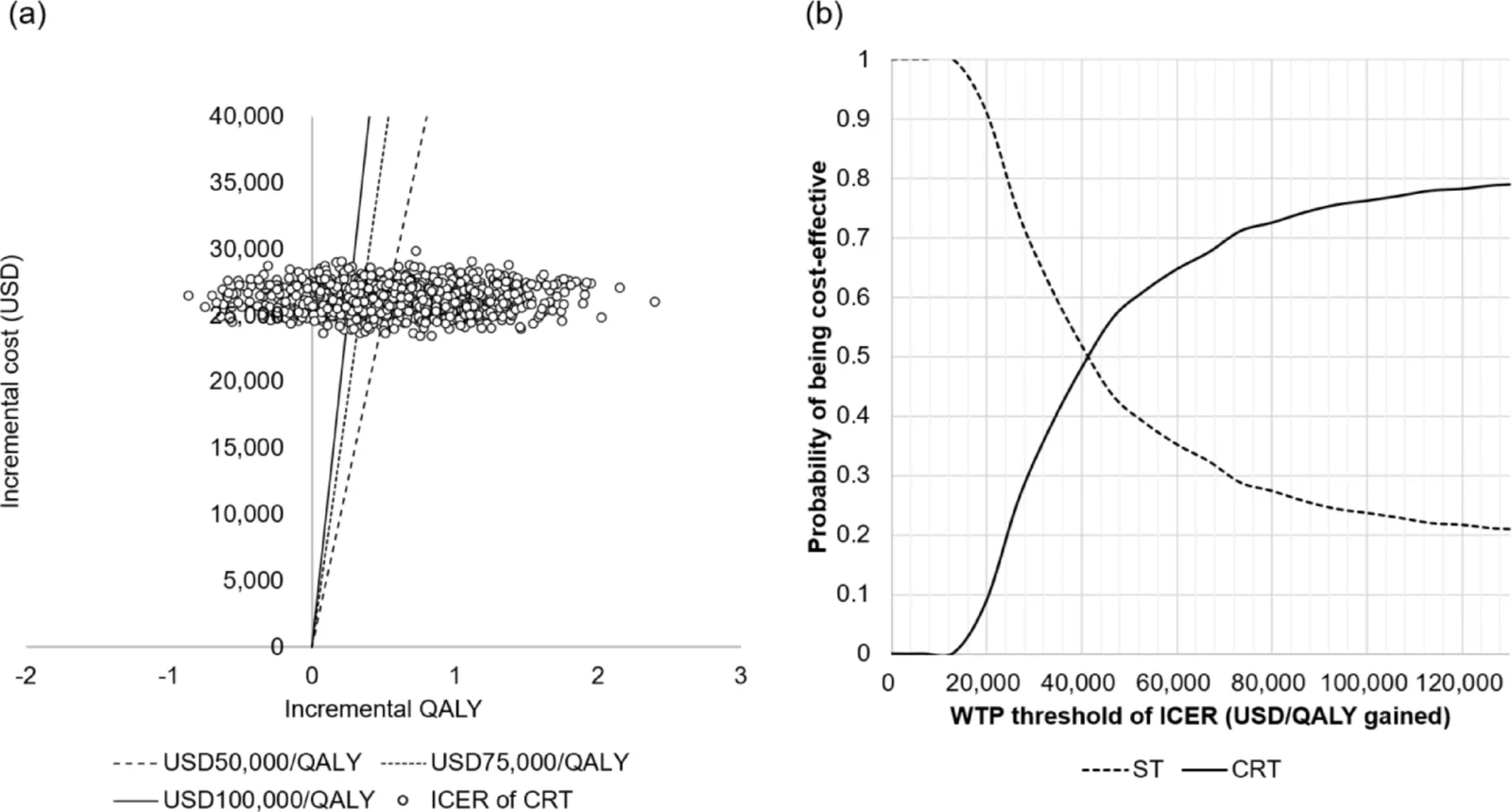
Probabilistic sensitivity analysis. Figure 4a shows a scatter plot, Fig. 4b shows the cost-effectiveness acceptability curves. *Abbreviations*: CRT: chemoradiotherapy, ICER: incremental cost-effectiveness ratio, USD: US Dollar, QALY: quality-adjusted life years, ST: surgical therapy, WTP: willingness-to-pay

**Fig. 5:**
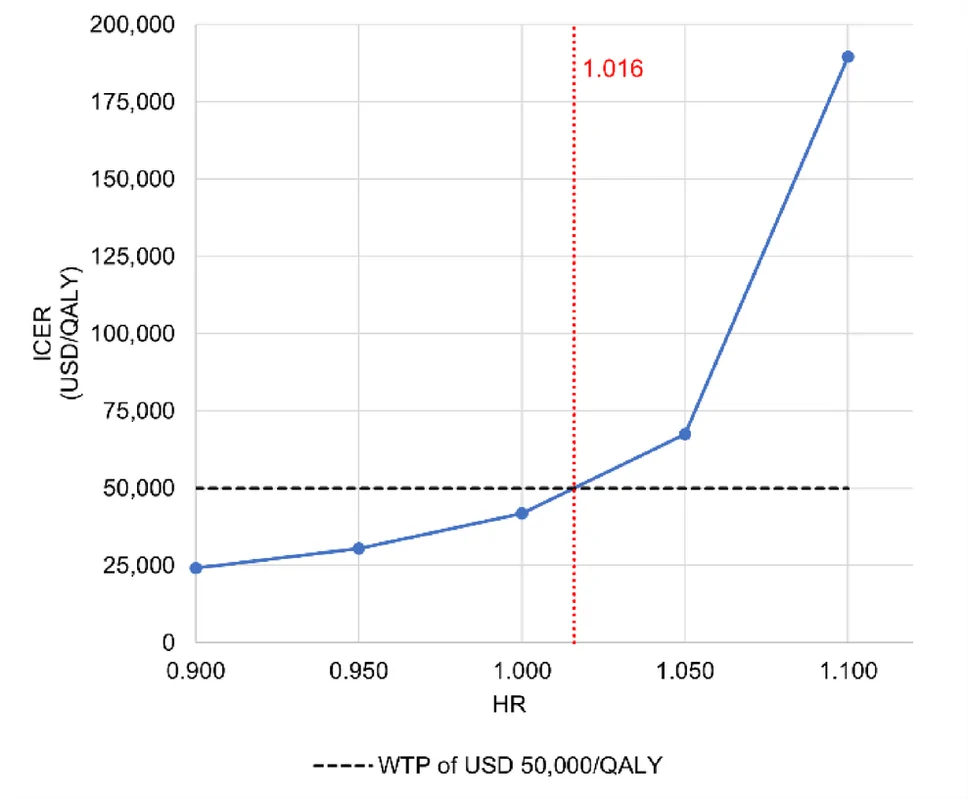
Sensitivity analysis for OS. Figure 5 shows the change in WTP varies according to the HR for OS in the ST group. When HR was set as 1.016, the WTP exceeded $50,000. *Abbreviations*: ICER: incremental cost-effectiveness ratio, HR: hazard ratio, USD: US Dollar, QALY: quality-adjusted life years, ST: surgical therapy, WTP: willingness-to-pay

The results of probabilistic sensitivity analysis are summarized in Fig. 4. The cost-effectiveness acceptability curve showed that CRT was more cost-effective than ST were estimated to be 59.3%, 67.5%, and 75.8%, when the acceptable values for ICER were $50,000, $75,000, and $100,000/QALY, respectively. The result of sensitivity analysis for OS was summarized in Table 4; Fig. 5. It showed that OS for ST group exceeded the lower bound of our ICER when the HR was set as 1.016. In the scenario analyses, the ICER increased to $56,751 in Scenario 1, in which age adjustment was applied (Supp Table 2). In Scenario 2, in which follow-up costs during the PFS period were adjusted, the ICER was $42,597 and remained below the prespecified threshold (Supp Table 3). Threshold analysis showed that the ICER exceeded the threshold when follow-up costs increased to 1.315 times in the CRT group and when they decreased to 0.526 times in the ST group (Supp Figs. 1 and 2). In Scenario 3, the addition of ICI during the PD period increased the incremental monthly cost up to $3289 and ICER reached $125,401, exceeding the upper limit prespecified (Supp Table 4).

**Table 4:** Sensitivity analysis of HR for OS

HR for OS in ST	Treatment	Cost (USD)	Incremental cost (USD)	QALY	Incremental QALY	ICER (USD/QALY)
0.900	ST	46,190		10.029		
	CRT	74,489	28,299	11.203	1.175	24,094
0.950	ST	47,156		10.306		
	CRT	74,489	27,333	11.203	0.897	30,470
1.000	ST	48,174		10.573		
	CRT	74,489	26,315	11.203	0.630	41,744
1.050	ST	49,245		10.829		
	CRT	74,489	25,244	11.203	0.374	67,502
1.100	ST	50,368		11.076		
	CRT	74,489	24,121	11.203	0.127	189,508

CRT: chemoradiotherapy, HR: Hazard ratio, ICER: incremental cost-effectiveness ratio, OS: Overall survival, QALY: quality-adjusted life years, ST: surgical treatment

## Discussion

In this study, we compared CRT to ST for stage I esophageal cancer from the perspective of the Japanese public healthcare payer. We estimated that the incremental cost was $26,313, the incremental QALY was 0.630, and ICER was $41,742/QALY.

In Japan, a system was initiated in 2019 to utilize the evidence of cost-effectiveness in the price adjustments of new medical technologies. Under this system, a rule was adopted to adjust the proportion of the calculated price of new medical technologies that corresponded to the additional premium, according to the level of ICER. For the price adjustment of items that required special consideration, three ICER thresholds were established before mentioned. Although technologies other than drugs and medical devices are currently not covered by this system, the minimum acceptable value for ICER is $50,0000/QALY, the point estimate-based result of $41,742/QALY obtained in this study showed that CRT was superior to ST in terms of cost-effectiveness. Although our sensitivity analysis indicates the superiority of CRT, the results are not robust due to several factors. First, regarding the difference in QALYs between the two groups, several articles [22–25] indicated that patients in the ST group may obtain improved QALYs because of less invasive treatments and the introduce of perioperative adjuvant therapy. Inomata et al. [22]. reported that several studies of minimally invasive esophagectomy indicated that early interventions such as perioperative rehabilitation within the Enhanced Recovery After Surgery framework can reduce various adverse events associated with ST [23–25]. In Japan, the use of robot-assisted minimally invasive surgery has been increasing since it became eligible for health insurance coverage in 2018 [26]. Comparisons of HRQOL have shown improvements even when compared with thoracoscopic surgery [27], and it is highly likely that QALYs will increase in the ST group. With respect to costs, although evidence from the United States indicates increased expenditures [28] and the procedure is categorized as cost-increasing in Japan, benefits such as shorter hospitalization durations and fewer complications [29] have been observed. Consequently, the overall impact on total costs remains a subject for further evaluation.

These findings suggest that the comparable significance of the improved QALYs between CRT and ST is not fixed. Additionally, it should be noted that Intensity-Modulated Radiation Therapy (IMRT) is being incorporated into the CRT and is anticipated to enhance QOL by reducing the adverse events. In both groups, the treatment procedures are becoming less invasive, which is likely to affect future estimates on incremental QALYs and ICERs. From a cost perspective, the introduction of IMRT may increase the initial treatment costs for the CRT group [30] and potentially alter the ICER.

We employed a utility analysis developed by Ariga et al. [8]. that was conducted for ethnic groups, treatment techniques, and public insurance similar to those in the JCOG0502 [5]. However, results of utility analysis of esophageal cancer treatments vary as mentioned [9, 10] and these results are inconsistent. Therefore, the construction of a model derived from multiple studies has the potential to yield divergent results. In JCOG0502 [5], the mean age was lower in the ST group, whereas in the Ariga et al. [8]. published the mean age was equal in both groups. To account for differences in ICER based on age, we conducted an additional scenario analysis. As a result, QALY for CRT group declined and the ICER increased by $56,751. These findings suggest that the superiority of the CRT group is not consistently robust. In addition, we assumed equivalent OS between the ST and CRT. However, as shown in Table 4; Fig. 5, when the OS for ST group was set at HR = 1.016, it exceeded the lower bound of the ICER we defined. If the OS for ST group is slightly better than that for CRT, the ST could potentially be considered more cost-effective.

Since 2018, ICI for recurrent esophageal cancer has been covered by public insurance in Japan and has been increasingly used in clinical practice. Given the high cost of ICI, we conducted an additional scenario analysis and found that the ICER increased to $125,401. However, this analysis simply added ICI costs to a model based on JCOG0502 [5] and Ariga et al. [8] and did not permit detailed modeling of OS, DFS, and QALYs associated with ICI. Although these results appear to reflect a higher recurrence rate in the CRT group compared with the ST group, the data using this study are insufficient to allow for a detailed cost-effectiveness modelling with ICI. Consequently, a dedicated cost-effectiveness analysis comparing the introduction of ICI in the ST and CRT groups remains an important area for future research.

While this study showed that CRT was superior in cost-effectiveness in this study, ST might be superior in cost-effectiveness influenced by various factors as mentioned above. This indicates that both ST and CRT are equally effective and cost-effective in the Japanese domestic insurance system. Therefore, treatment decisions can be made based on shared decision-making with the patient.

The novelty of this study was that it used the JMDC claims database to evaluate the cost-effectiveness of CRT and ST for esophageal cancer. The JMDC claims database is the largest commercial epidemiological receipt database in Japan. Using this database, we were able to estimate ICERs that considered the real-world setting in Japan. It is also significant that we applied the EQ-5D-5 L mapping algorithm to the EORTC-QLQ30 data to estimate utility weights that reflected the preferences of the general population in Japan for various health conditions of esophageal cancer patients. Our approaches could be applied to health economic evaluations of patients with other types of cancer.

This study has several limitations. At first, the JMDC data only included receipt data and did not provide clinical information on patients, such as stage. Therefore, the analysis could not be limited to stage I patients. The cost of maintenance chemotherapy following CRT for stage II–III patients may be perceived as an additional factor to consider. Second, clinical events such as progression depended on analytical definitions in this study. The analysis utilized surrogate triggers, such as re-initiation of surgery, radiation therapy after surgery, or chemotherapy occurring after completion of the initial therapy. Third, endoscopic treatments and photodynamic therapy could not be included as triggers because of the lack of explicit data. Fourth, the survival probabilities were based on the best visual fit and did not incorporate individual patient data from JCOG0502 [5]. Therefore, the accuracy of specific survival probabilities may be lower than if individual patient data were used. Fifth, we found it difficult to develop a model that incorporates the facility-specific three coefficients—base coefficients, functional evaluation coefficients I and II—in Japan’s health insurance, and we were unable to develop prescription medicine models depending on each facility or patient. At last, the insurance medical fee in Japan revises every two years, these changes affect above all and creating uncertainty on cost parameters.

In conclusion, CRT for stage I esophageal cancer in Japan is likely to be more cost-effective than ST. Although further investigation of resource consumption, HRQOL, and clinical benefits in Japanese patients is needed, our approach is expected to support clinical and policy decision making.

## Electronic Supplementary Material

Below is the link to the electronic supplementary material.

Supplementary Material

## Data Availability

Research data are not available at this time.
