# Supplementary material for: Cost-effectiveness analysis of chemoradiotherapy compared to surgery in patients with stage I esophageal squamous cell carcinoma in Japan
Source: Int J Clin Oncol. 2026 Jul 23;31(10):2394–404. doi: 10.1007/s10147-026-03145-5 (PMC13615109; doi:10.1007/s10147-026-03145-5)
Supplement: Supplementary file 1 [file 10147_2026_3145_MOESM1_ESM.docx]

**Supplementary Fig. 1:** Threshold analysis of PFS costs in CRT group. The threshold analysis demonstrated that when PFS costs in the CRT group increase by a factor of 1.315, the threshold of USD 50,000 per QALY is exceeded**.**

Abbreviations: CRT: chemoradiotherapy, USD: US Dollar, PFS: progression-free survival, QALY: quality-adjusted life years.

**Supplementary Fig. 2:** Threshold analysis of PFS costs in ST group. The threshold analysis demonstrated that when PFS costs in the ST group decrease to a factor of 0.526, the threshold of USD 50,000 per QALY is exceeded.

Abbreviations: USD: US Dollar, PFS: progression-free survival, QALY: quality-adjusted life years, ST: surgical therapy.

| **Supplementary Table 1. Parameter settings for scenario analyses** | | |
| --- | --- | --- |
|  | Estimate | Reference |
| For scenario analysis 1 |  |  |
| Age dependent utility weights |  | Shiroiwa et al [16] |
| Start age |  |  |
| ST group | 62 | JCOG0502 [5] |
| CRT group | 65 | JCOG0502 [5] |
| Proportion of male |  |  |
| ST group | 82.80% | JCOG0502 [5] |
| CRT group# | 88.10% | JCOG0502 [5] |
|  |  |  |
| For scenario analysis 2 |  |  |
| Medical cost in PFS state after esophagectomy (/month) |  |  |
| 1st year | 138 |  |
| 2nd year | 123 |  |
| 3rd year | 123 |  |
| 4th year | 123 |  |
| 5th year | 123 |  |
| Subsequent year | 55 |  |
| Medical cost in PFS state with esophagus preservation (/month) |  |  |
| 1st year | 328 |  |
| 2nd year | 219 |  |
| 3rd year | 219 |  |
| 4th year | 219 |  |
| 5th year | 219 |  |
| Subsequent year | 55 |  |
|  |  |  |
| For scenario analysis 3 |  |  |
| Drug cost of ICI in PD state (USD/month) | 3,289 |  |

**Supplementary Table 2. Results of scenario analysis1**

|  | ST | CRT | Incremental  (vs ST) |
| --- | --- | --- | --- |
| Life year | 15.323 | 15.323 | 0.000 |
| Duration in PFS after esophagectomy (year) | 14.970 | 0.000 | -14.970 |
| Duration in PFS with esophagus preservation (year) | 0.000 | 13.392 | 13.392 |
| Duration in PD after esophagectomy (year) | 0.354 | 0.445 | 0.091 |
| Duration in PD with esophagus preservation (year) | 0.000 | 1.487 | 1.487 |
| QALY | 10.205 | 10.668 | 0.464 |
| Total cost (USD) | 48,174 | 74,489 | 26,315 |
| Initial treatment cost | 20,163 | 7,262 | -12,901 |
| Medical cost in PFS after esophagectomy | 10,989 | 0 | -10,989 |
| Medical cost in PFS with esophagus preservation | 0 | 16,538 | 16,538 |
| Medical cost in PD after esophagectomy | 8,010 | 9,671 | 1,660 |
| Cost of esophagectomy in CRT group | 0 | 1,066 | 1,066 |
| Medical cost in PD with esophagus preservation | 0 | 30,940 | 30,940 |
| ICER (USD/QALY gained) |  |  | 56,751 |

**Supplementary Table 3. Results of scenario analysis2**

|  | ST | CRT | Incremental  (vs ST) |
| --- | --- | --- | --- |
| Life year | 15.323 | 15.323 | 0.000 |
| Duration in PFS after esophagectomy (year) | 14.970 | 0.000 | -14.970 |
| Duration in PFS with esophagus preservation (year) | 0.000 | 13.392 | 13.392 |
| Duration in PD after esophagectomy (year) | 0.354 | 0.445 | 0.091 |
| Duration in PD with esophagus preservation (year) | 0.000 | 1.487 | 1.487 |
| QALY | 10.573 | 11.203 | 0.630 |
| Total cost (USD) | 49,659 | 76,511 | 26,852 |
| Initial treatment cost | 20,163 | 7,262 | -12,901 |
| Medical cost in PFS after esophagectomy | 12,474 | 0 | -12,474 |
| Medical cost in PFS with esophagus preservation | 0 | 18,560 | 18,560 |
| Medical cost in PD after esophagectomy | 8,010 | 9,671 | 1,660 |
| Cost of esophagectomy in CRT group | 0 | 1,066 | 1,066 |
| Medical cost in PD with esophagus preservation | 0 | 30,940 | 30,940 |
| ICER (USD/QALY gained) |  |  | 42,597 |

**Supplementary Table 4. Results of scenario analysis3**

|  | ST | CRT | Incremental  (vs ST) |
| --- | --- | --- | --- |
| Life year | 15.323 | 15.323 | 0.000 |
| Duration in PFS after esophagectomy (year) | 14.970 | 0.000 | -14.970 |
| Duration in PFS with esophagus preservation (year) | 0.000 | 13.392 | 13.392 |
| Duration in PD after esophagectomy (year) | 0.354 | 0.445 | 0.091 |
| Duration in PD with esophagus preservation (year) | 0.000 | 1.487 | 1.487 |
| QALY | 10.573 | 11.203 | 0.630 |
| Total cost (USD) | 61,132 | 140,182 | 79,050 |
| Initial treatment cost | 20,163 | 7,262 | -12,901 |
| Medical cost in PFS after esophagectomy | 10,989 | 0 | -10,989 |
| Medical cost in PFS with esophagus preservation | 0 | 16,538 | 16,538 |
| Medical cost in PD after esophagectomy | 20,969 | 25,314 | 4,346 |
| Cost of esophagectomy in CRT group | 0 | 1,066 | 1,066 |
| Medical cost in PD with esophagus preservation | 0 | 80,990 | 80,990 |
| ICER (USD/QALY gained) |  |  | 125,401 |
